# Supplementary material for: Diet Quality, Microbial Lignan Metabolites, and Cardiometabolic Health among US Adults
Source: Nutrients. 2023 Mar 15;15(6):1412. doi: 10.3390/nu15061412 (PMC10054147; doi:10.3390/nu15061412)
Supplement: Supplementary file 1 [file nutrients-15-01412-s001.zip › nutrients-2235853-supplementary.pdf]

Supplementary Material

Table of Contents

Page

I. **Supplementary Figures**

Participant Flowchart 3
Associations of Triglycerides with HEI, Enterolignans, and Energy Intake 4
Associations of LDL Cholesterol with HEI, Enterolignans, and Energy Intake 5
Associations of HDL Cholesterol with HEI, Enterolignans, and Energy Intake 6
Associations of Total-C with HEI, Enterolignans, and Energy Intake 7
Associations of Cardiometabolic Health Outcomes for Males 8
Associations of Cardiometabolic Health Outcomes for Females 9
Associations of Insulin with HEI, Enterolignans, and Energy Intake 10
Associations of OGTT with HEI, Enterolignans, and Energy Intake 11
Associations of Glucose with HEI, Enterolignans, and Energy Intake 12
Associations of HbA1c with HEI, Enterolignans, and Energy Intake 13
Associations of Adiposity with HEI, Enterolignans, and Energy Intake 14
Associations of Systolic Blood Pressure with HEI, Enterolignans, and Energy Intake. 15
Associations of Diastolic Blood Pressure with HEI, Enterolignans, and Energy Intake 16

II. **Supplementary Tables**

Model Coefficients for Varying Levels of Interaction for Cardiometabolic Health, Diet Quality, and Microbial Lignan Metabolites. 17
Model Coefficients for Waist Circumference Sensitivity Analysis. 18
Male Stratified Model Coefficients for Cardiometabolic Health, Diet Quality, and Microbial Lignan Metabolites. 19
Female Stratified Model Coefficients for Cardiometabolic Health, Diet Quality, and Microbial Lignan Metabolites. 20
Model Coefficients for Cardiometabolic Health, Diet Quality, and Microbial Lignan Metabolites with Interaction by Sex. 21

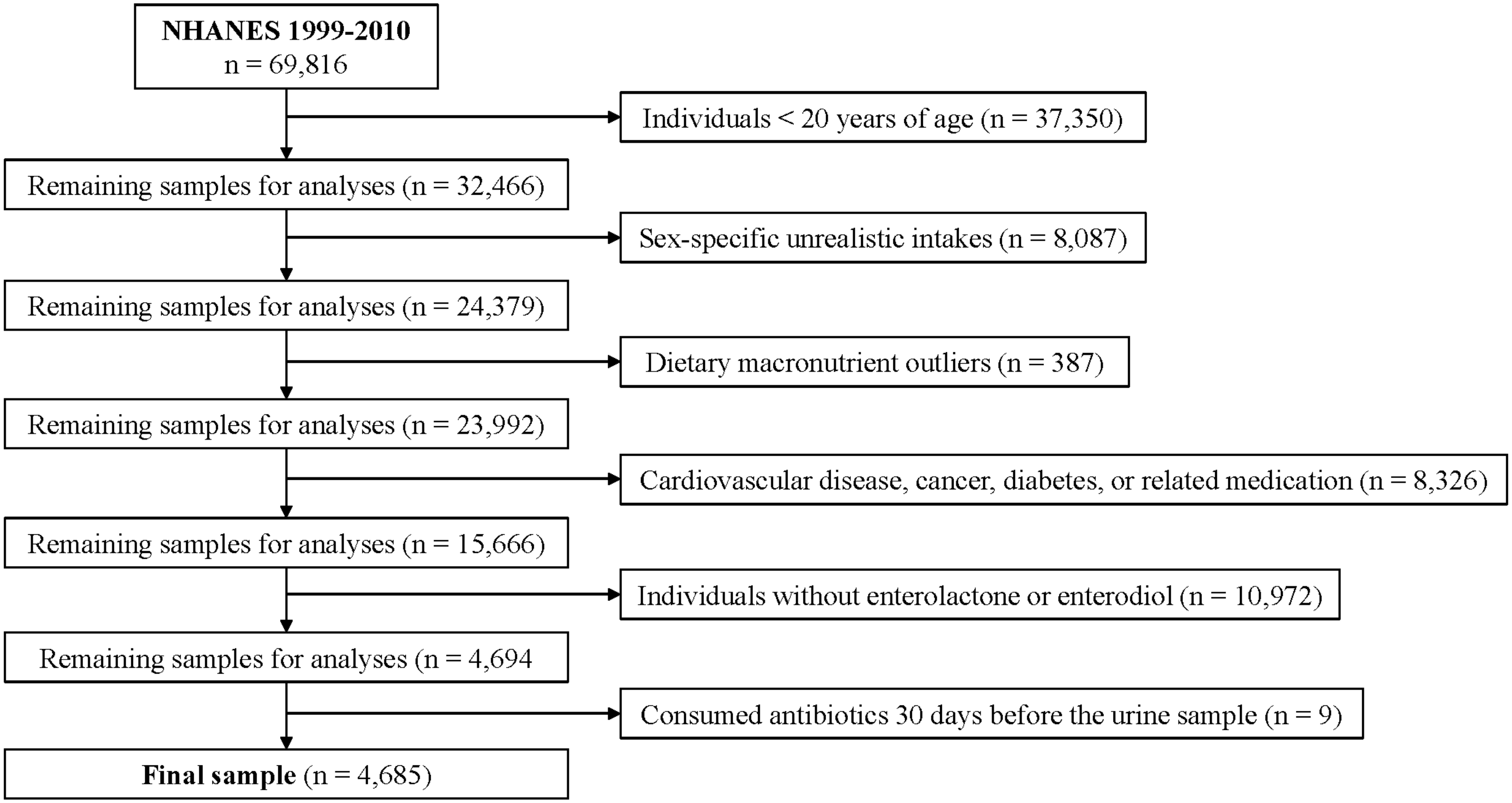

Figure S1. Participant Flow Chart.

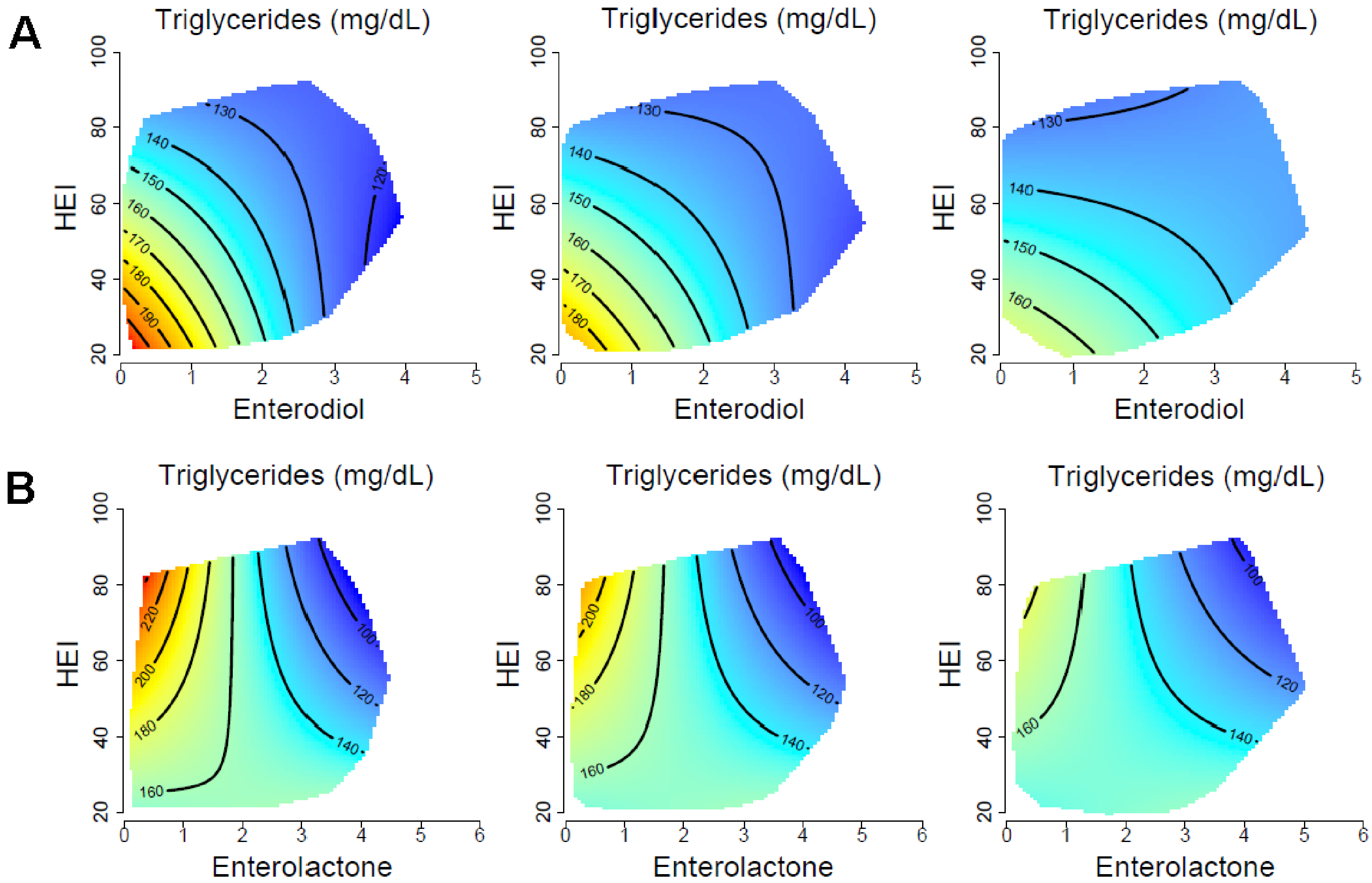

Figure S2. Associations of Triglycerides with HEI, Enterolignans, and Energy Intake. Response surfaces show the associations of triglycerides with HEI, enterodiol (A), enterolactone (B), and total energy intake. Enterolactone and enterodiol are presented as μmol/L (log-transformed). The outcome of each response surface is shown at the top of the plot with warmer colors denoting higher values and cooler colors denoting lower values. Response surfaces are predicted at the 25th, 50th, and 75th percentile of total energy intake from left to right and have been adjusted for age, sex, household income, BMI, and physical activity.

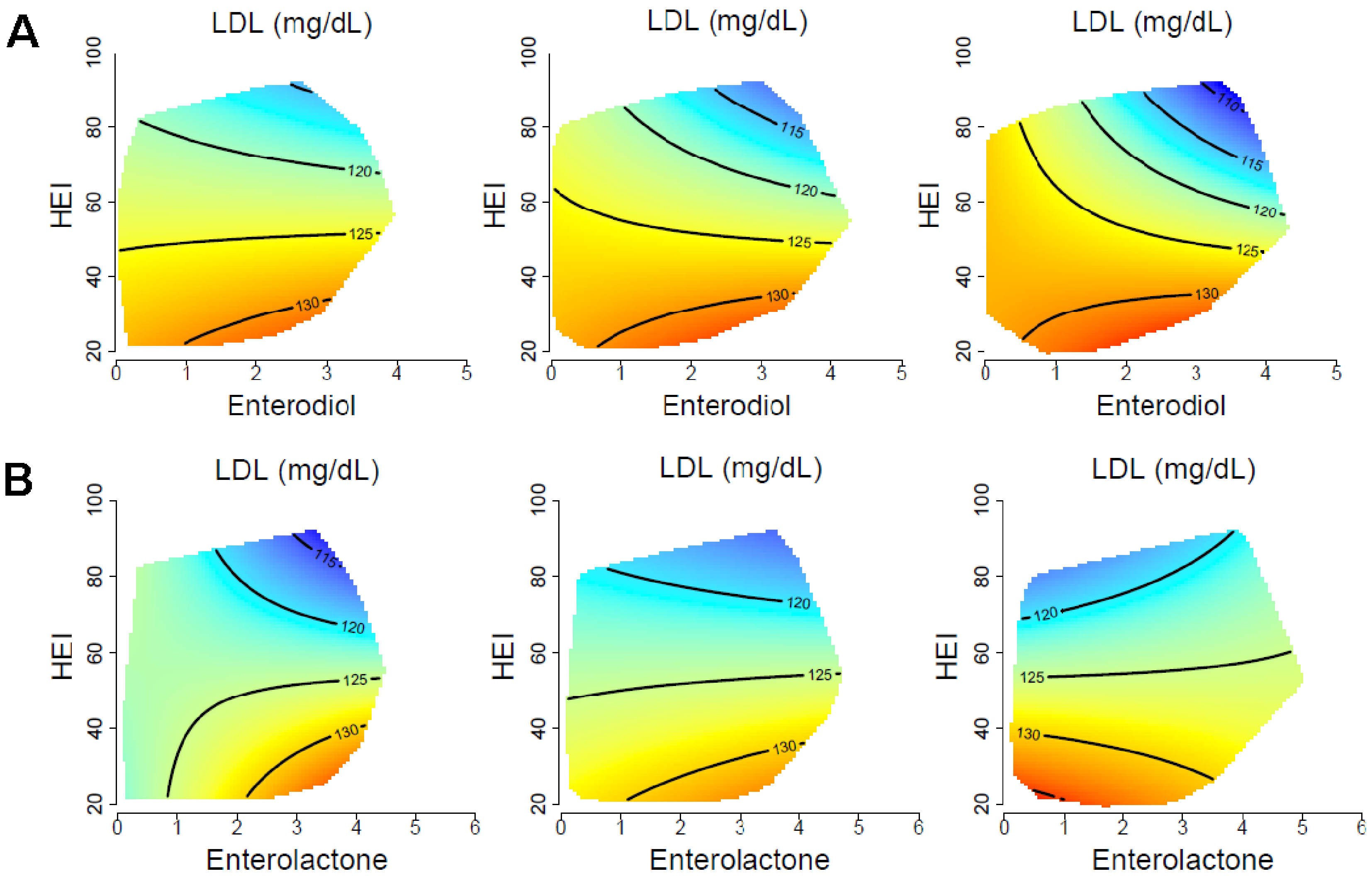

Figure S3. Associations of LDL Cholesterol with HEI, Enterolignans, and Energy Intake. Response surfaces show the associations of LDL cholesterol with HEI, enterodiol (A), enterolactone (B), and total energy intake. Enterolactone and enterodiol are presented as µmol/L (log-transformed). The outcome of each response surface is shown at the top of the plot with warmer colors denoting higher values and cooler colors denoting lower values. Response surfaces are predicted at the 25th, 50th, and 75th percentile of total energy intake from left to right and have been adjusted for age, sex, household income, BMI, and physical activity.

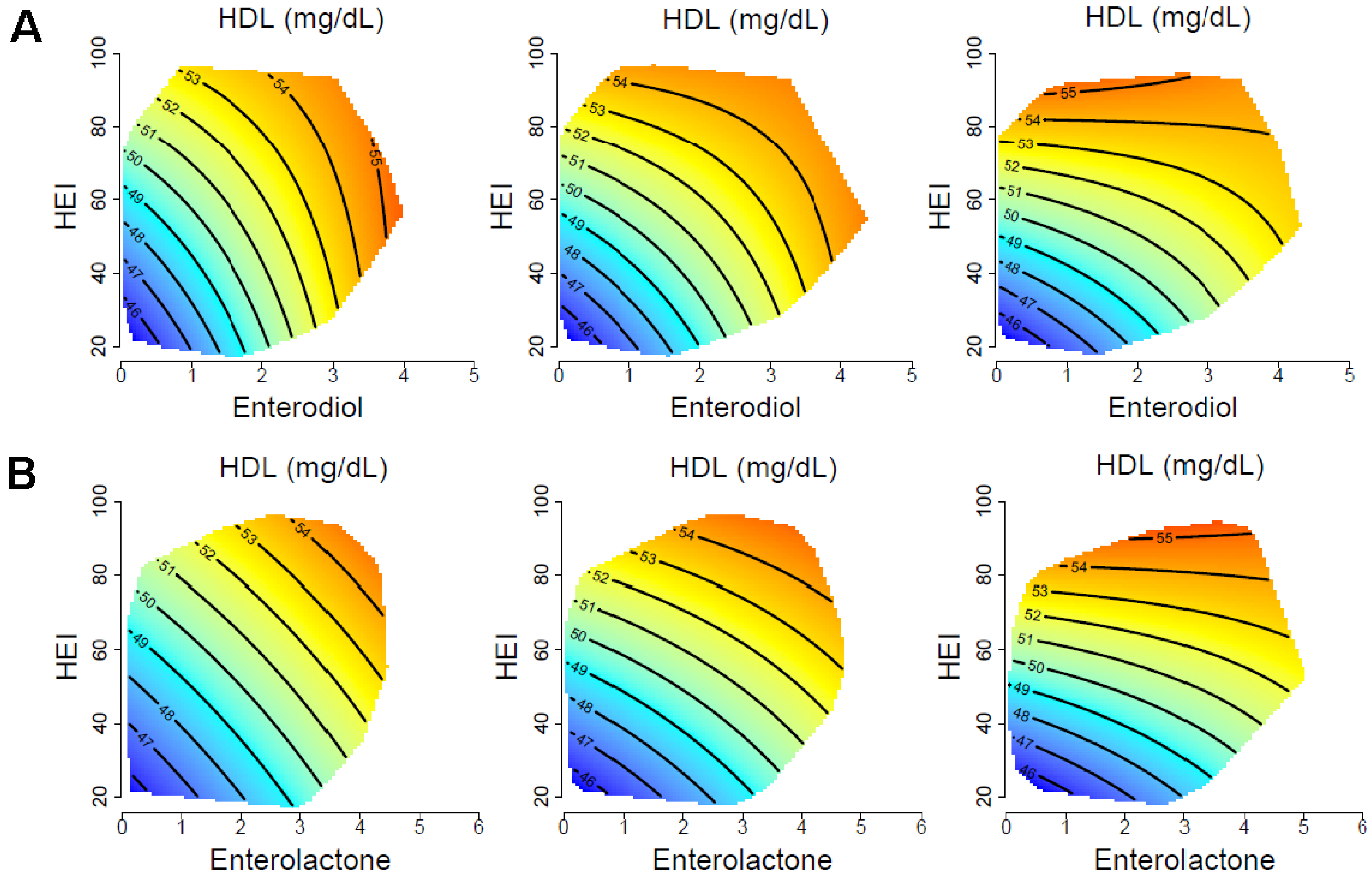

Figure S4. Associations of HDL Cholesterol with HEI, Enterolignans, and Energy Intake. Response surfaces show the associations of HDL cholesterol with HEI, enterodiol (A), enterolactone (B), and total energy intake. Enterolactone and enterodiol are presented as µmol/L (log-transformed). The outcome of each response surface is shown at the top of the plot with warmer colors denoting higher values and cooler colors denoting lower values. Response surfaces are predicted at the 25th, 50th, and 75th percentile of total energy intake from left to right and have been adjusted for age, sex, household income, BMI, and physical activity.

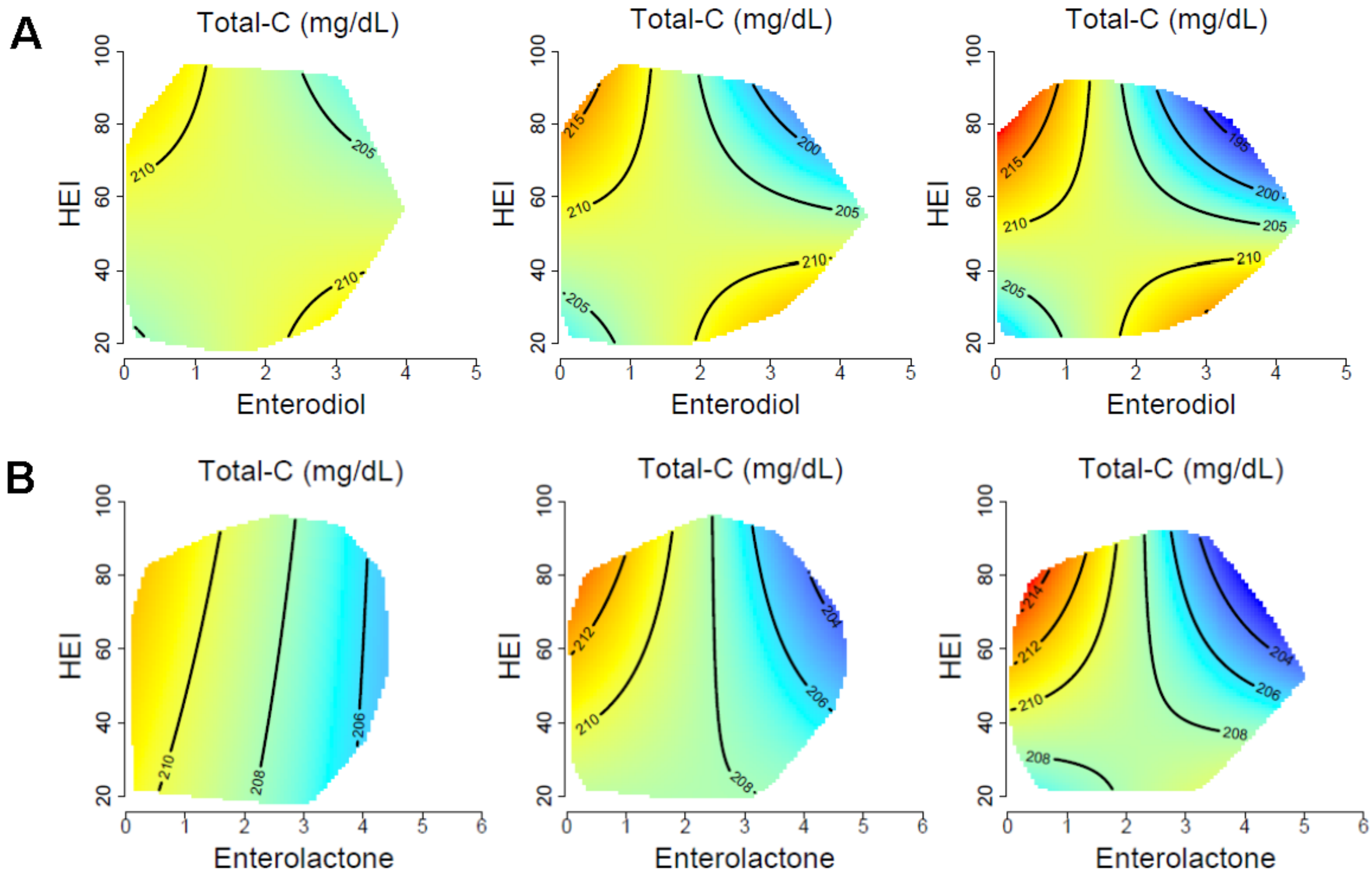

Figure S5. Associations of Total-C with HEI, Enterolignans, and Energy Intake. Response surfaces show the associations of Total-C with HEI, enterodiol (A), enterolactone (B), and total energy intake. Enterolactone and enterodiol are presented as µmol/L (log-transformed). The outcome of each response surface is shown at the top of the plot with warmer colors denoting higher values and cooler colors denoting lower values. Response surfaces are predicted at the 25th, 50th, and 75th percentile of total energy intake from left to right and have been adjusted for age, sex, household income, BMI, and physical activity.

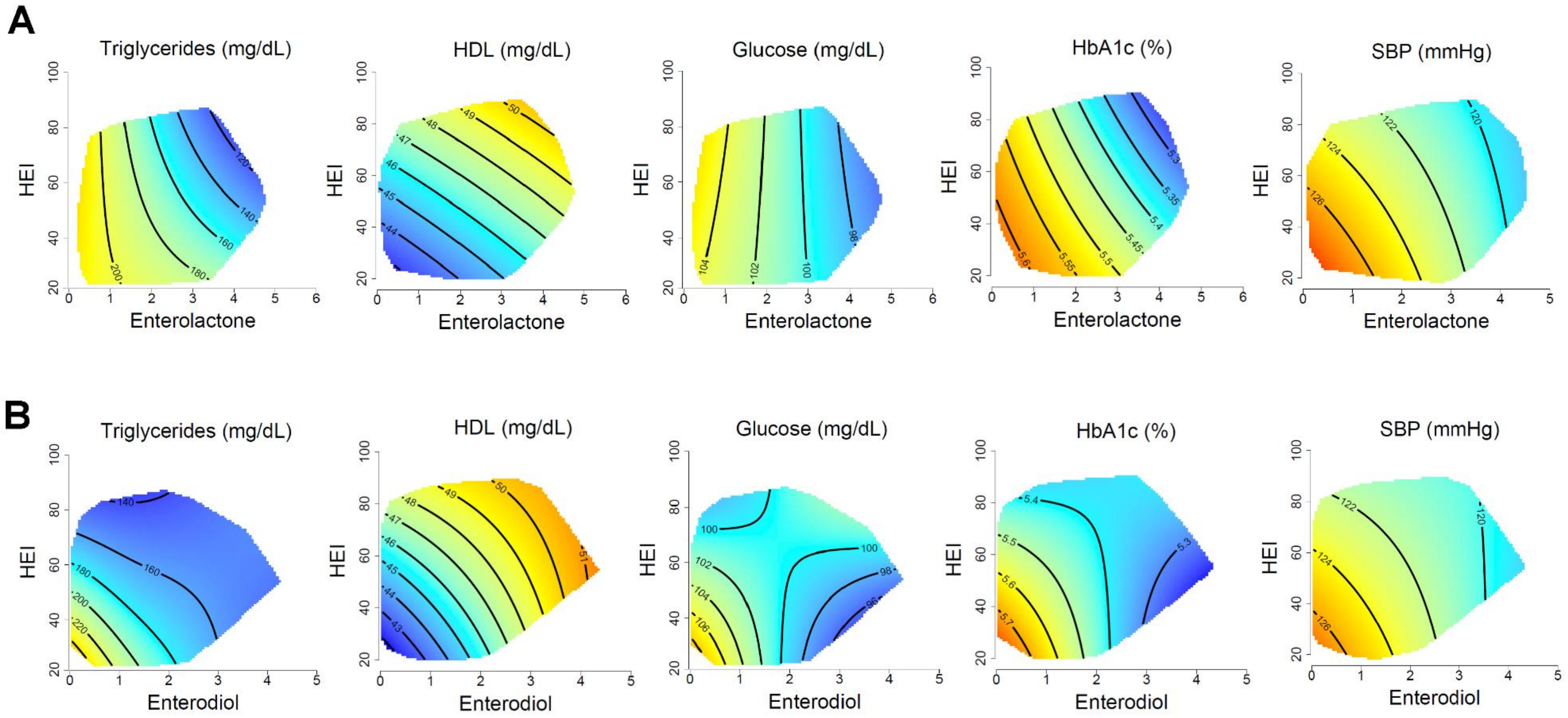

Figure S6. Associations of Cardiometabolic Health Outcomes for Males. Response surfaces show the associations of significant cardiometabolic health markers with HEI, enterolactone (A), enterodiol (B) while holding total energy intake constant at the 50th percentile. Enterolactone and enterodiol are presented as µmol/L (log-transformed). The outcome of each response surface is shown at the top of the plot with warmer colors denoting higher values and cooler colors denoting lower values. Response surfaces are predicted at the 25th, 50th, and 75th percentile of total energy intake from left to right and have been adjusted for age, household income, BMI, and physical activity.

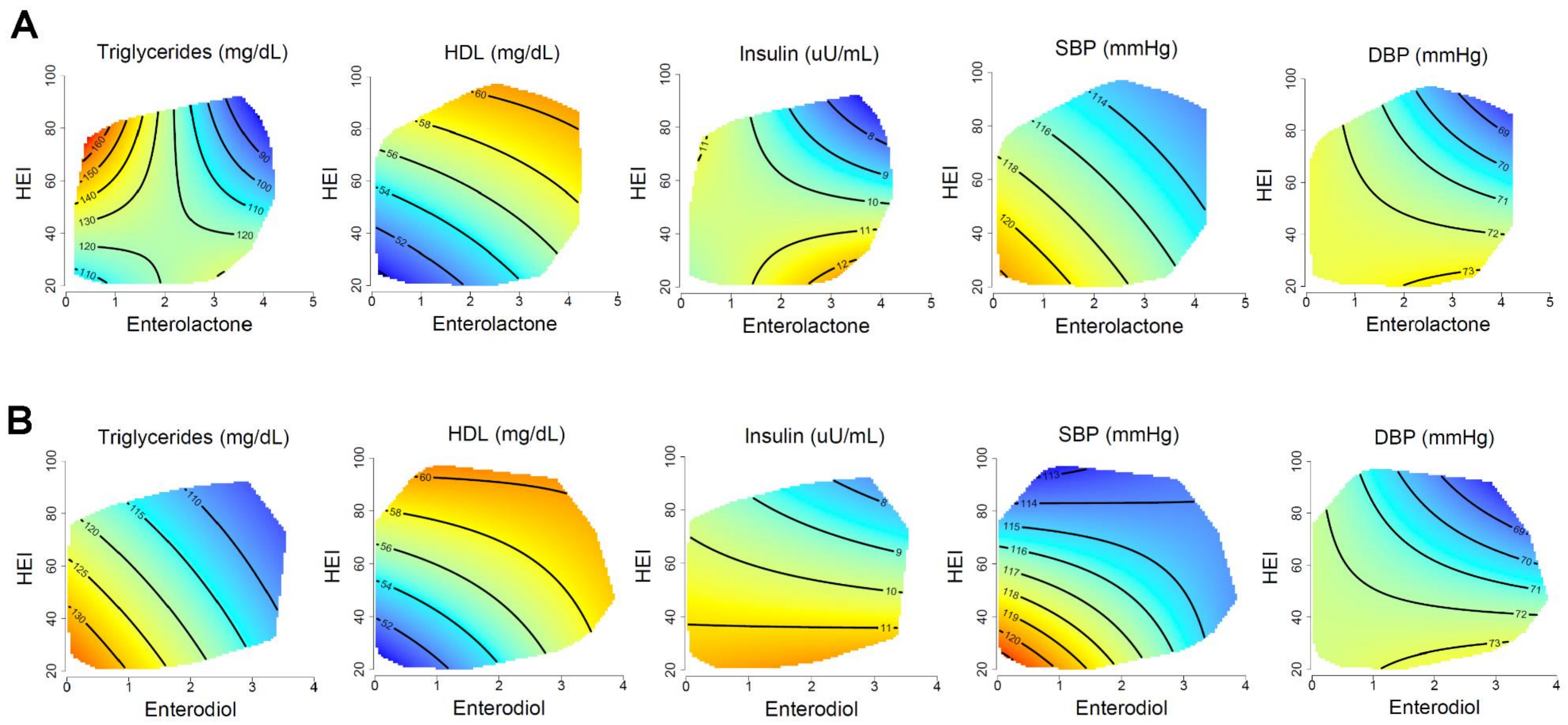

Figure S7. Associations of Cardiometabolic Health Outcomes for Females. Response surfaces show the associations of significant cardiometabolic health markers with HEI, enterolactone (A), enterodiol (B) while holding total energy intake constant at the 50th percentile. Enterolactone and enterodiol are presented as µmol/L (log-transformed). The outcome of each response surface is shown at the top of the plot with warmer colors denoting higher values and cooler colors denoting lower values. Response surfaces are predicted at the 25th, 50th, and 75th percentile of total energy intake from left to right and have been adjusted for age, household income, BMI, and physical activity.

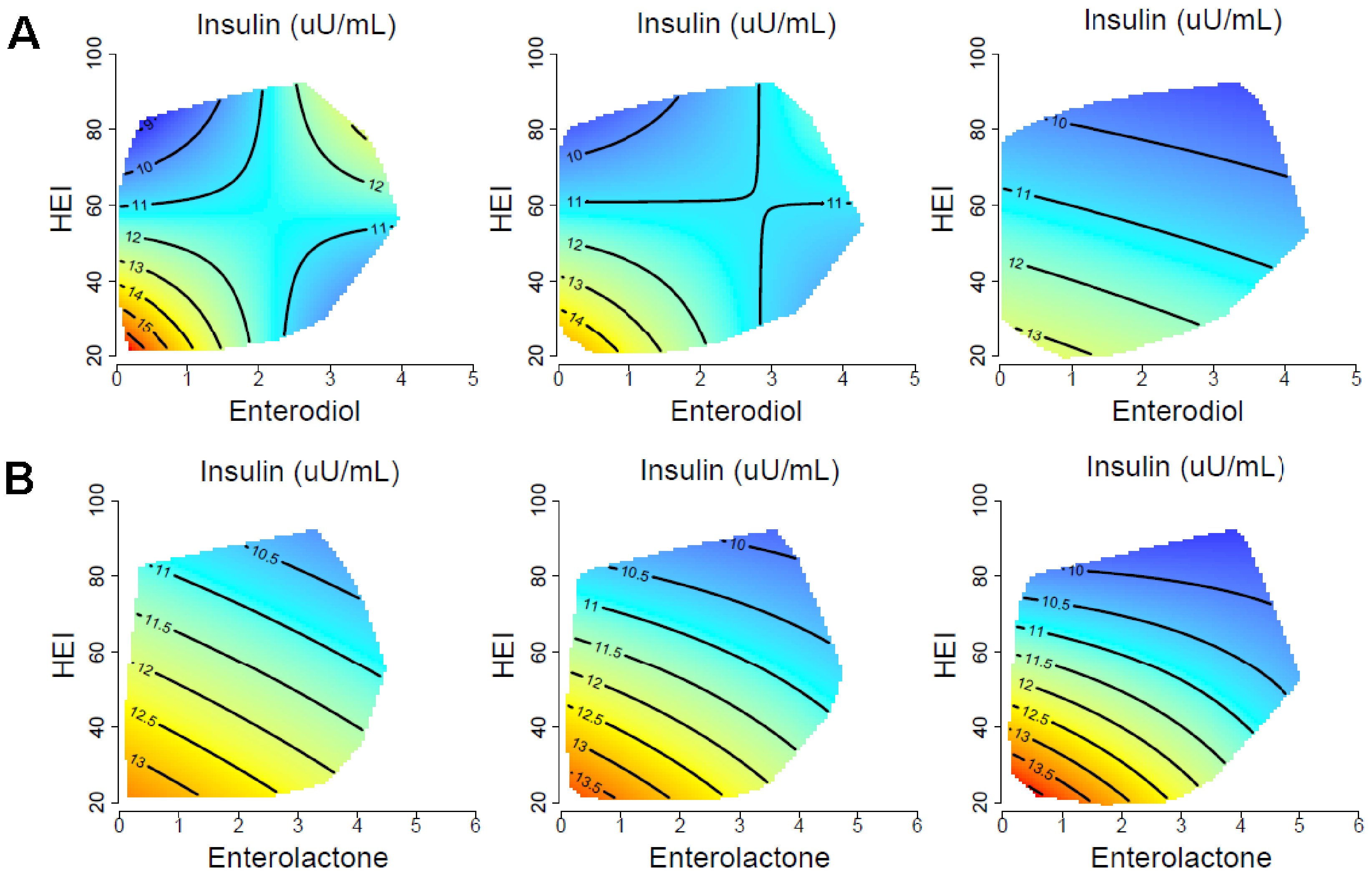

Figure S8. Associations of Insulin with HEI, Enterolignans, and Energy Intake. Response surfaces show the associations of Insulin with HEI, enterodiol (A), enterolactone (B), and total energy intake. Enterolactone and enterodiol are presented as µmol/L (log-transformed). The outcome of each response surface is shown at the top of the plot with warmer colors denoting higher values and cooler colors denoting lower values. Response surfaces are predicted at the 25th, 50th, and 75th percentile of total energy intake from left to right and have been adjusted for age, sex, household income, BMI, and physical activity.

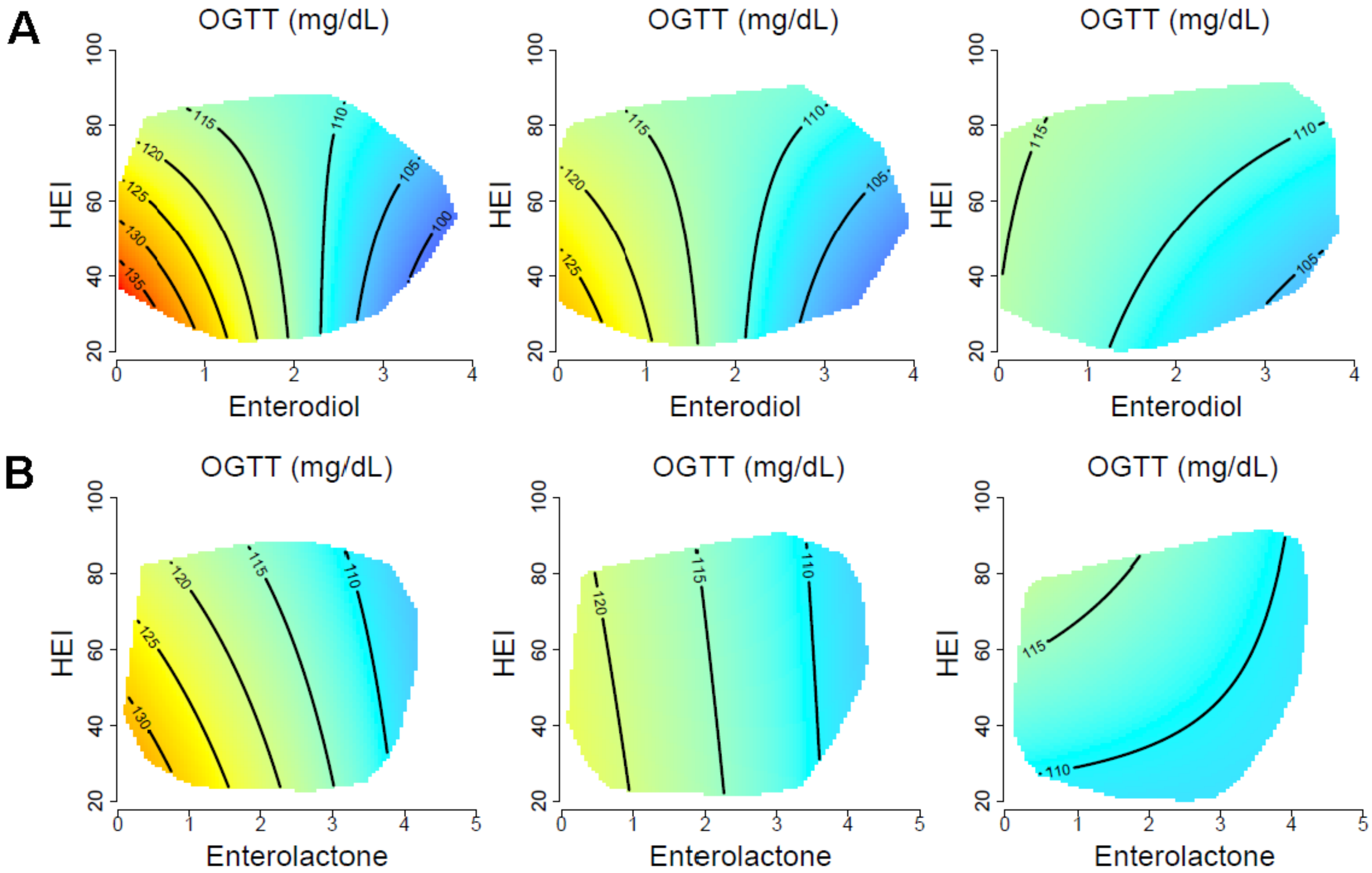

Figure S9. Associations of OGTT with HEI, Enterolignans, and Energy Intake. Response surfaces show the associations of OGTT with HEI, enterodiol (A), enterolactone (B), and total energy intake. Enterolactone and enterodiol are presented as µmol/L (log-transformed). The outcome of each response surface is shown at the top of the plot with warmer colors denoting higher values and cooler colors denoting lower values. Response surfaces are predicted at the 25th, 50th, and 75th percentile of total energy intake from left to right and have been adjusted for age, sex, household income, BMI, and physical activity.

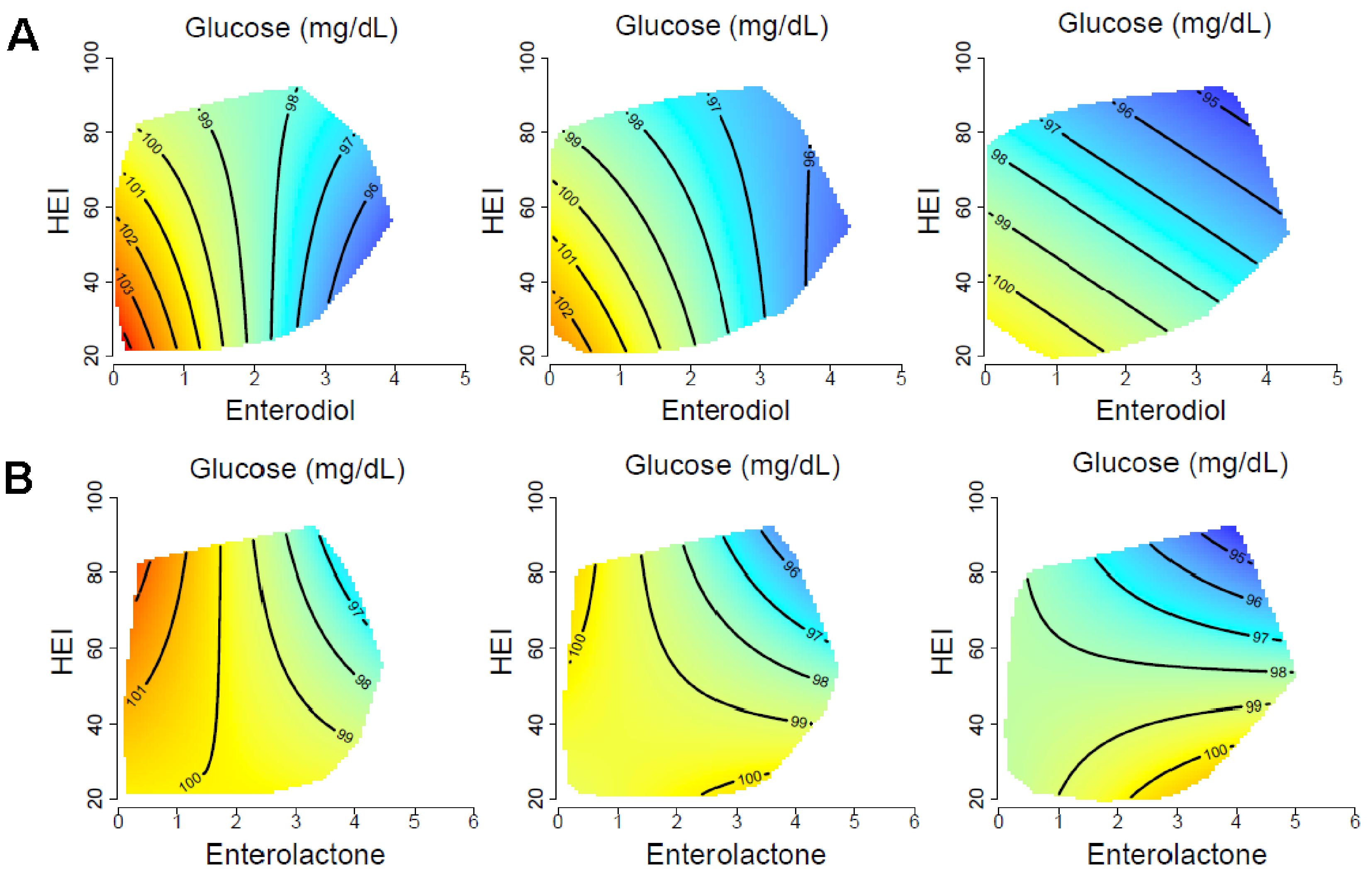

Figure S10. Associations of Glucose with HEI, Enterolignans, and Energy Intake. Response surfaces show the associations of glucose with HEI, enterodiol (A), enterolactone (B), and total energy intake. Enterolactone and enterodiol are presented as µmol/L (log-transformed). The outcome of each response surface is shown at the top of the plot with warmer colors denoting higher values and cooler colors denoting lower values. Response surfaces are predicted at the 25th, 50th, and 75th percentile of total energy intake from left to right and have been adjusted for age, sex, household income, BMI, and physical activity.

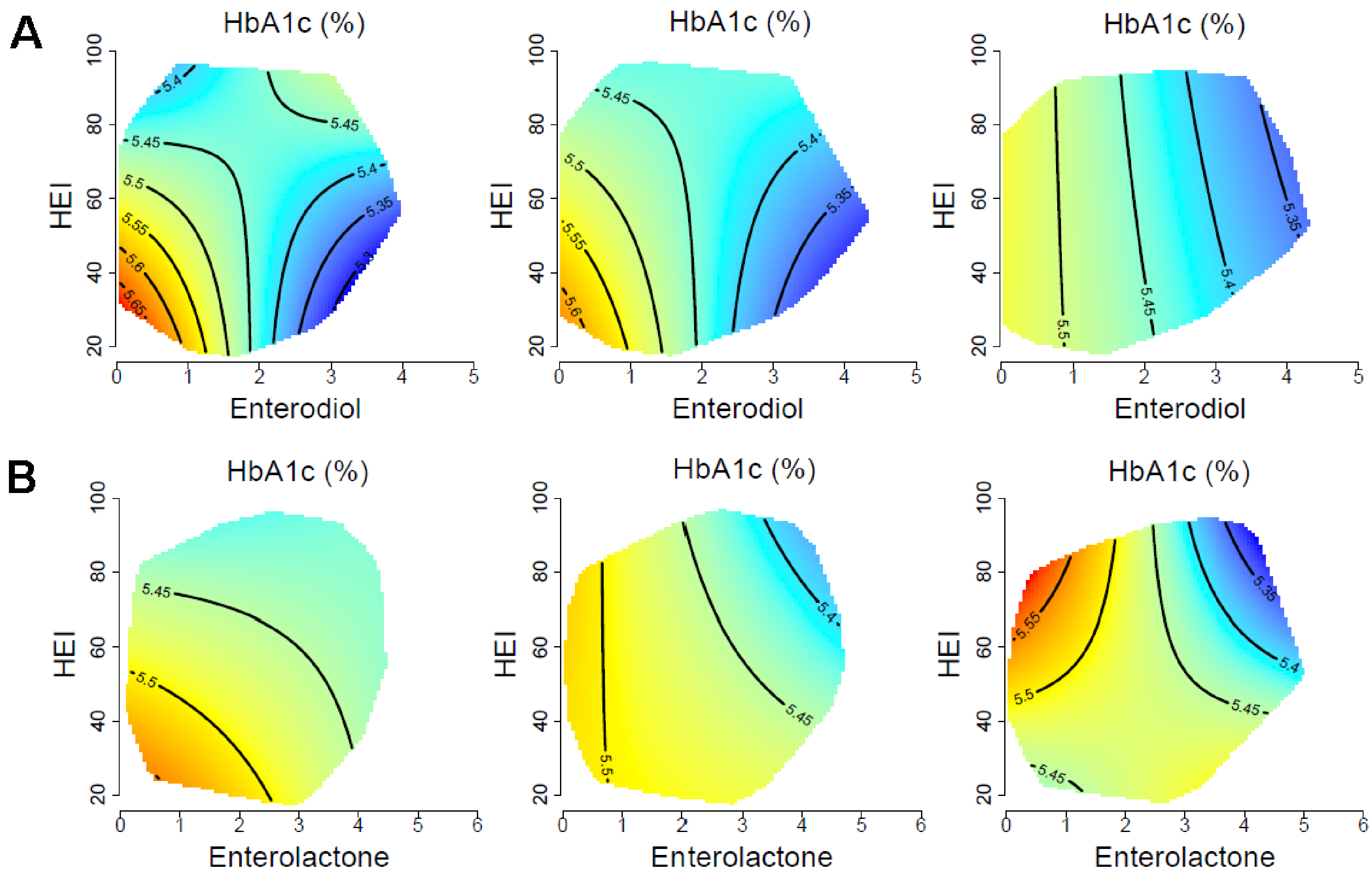

Figure S11. Associations of HbA1c with HEI, Enterolignans, and Energy Intake. Response surfaces show the associations of HbA1c with HEI, enterodiol (A), enterolactone (B), and total energy intake. Enterolactone and enterodiol are presented as μmol/L (log-transformed). The outcome of each response surface is shown at the top of the plot with warmer colors denoting higher values and cooler colors denoting lower values. Response surfaces are predicted at the 25th, 50th, and 75th percentile of total energy intake from left to right and have been adjusted for age, sex, household income, BMI, and physical activity.

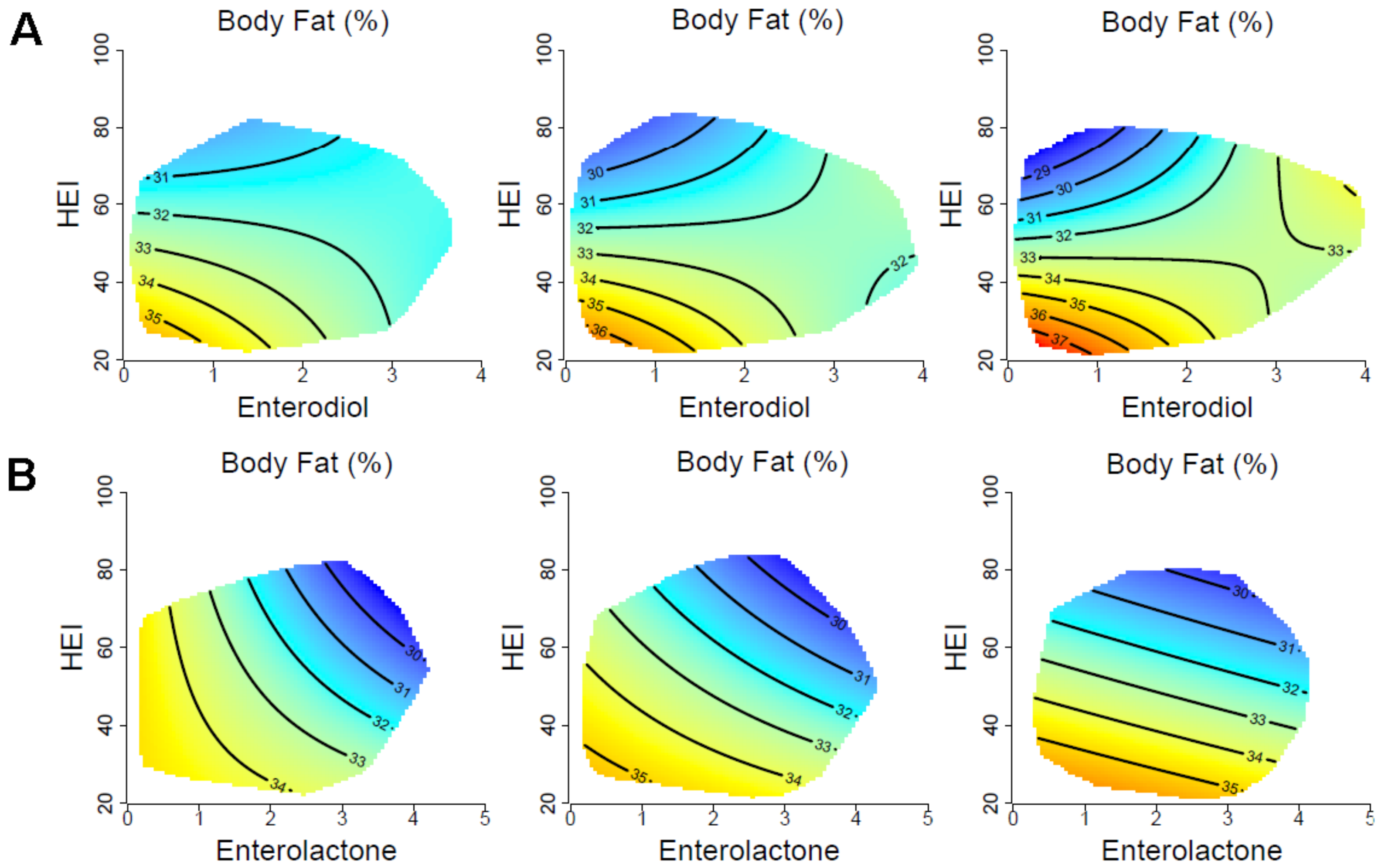

Figure S12. Associations of Adiposity with HEI, Enterolignans, and Energy Intake. Response surfaces show the associations of diastolic adiposity with HEI, enterodiol (A), enterolactone (B), and total energy intake. Enterolactone and enterodiol are presented as µmol/L (log-transformed). The outcome of each response surface is shown at the top of the plot with warmer colors denoting higher values and cooler colors denoting lower values. Response surfaces are predicted at the 25th, 50th, and 75th percentile of total energy intake from left to right and have been adjusted for age, sex, household income, BMI, and physical activity.

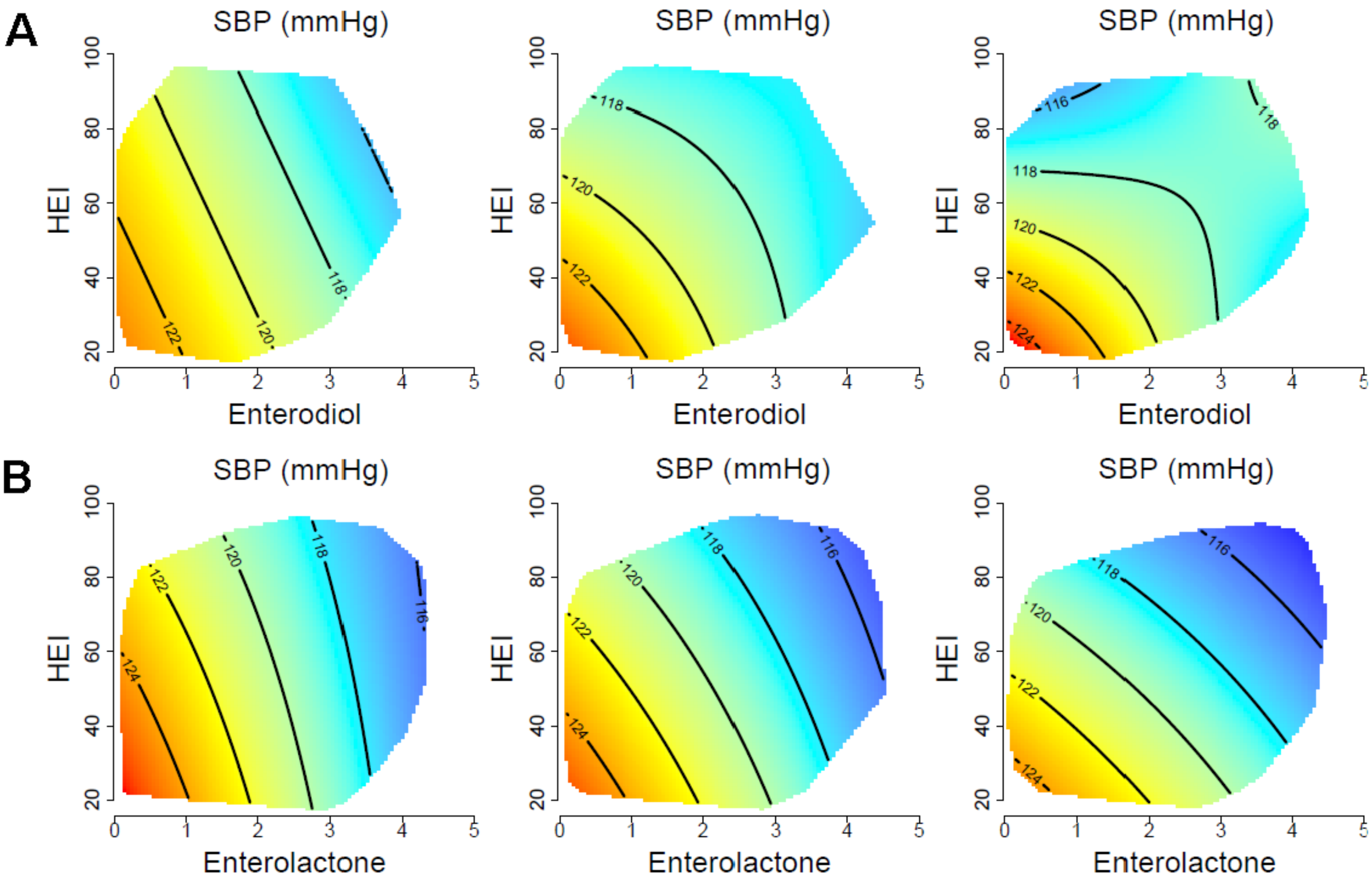

Figure S13. Associations of Systolic Blood Pressure with HEI, Enterolignans, and Energy Intake. Response surfaces show the associations of systolic blood pressure with HEI, enterodiol (A), enterolactone (B), and total energy intake. Enterolactone and enterodiol are presented as µmol/L (log-transformed). The outcome of each response surface is shown at the top of the plot with warmer colors denoting higher values and cooler colors denoting lower values. Response surfaces are predicted at the 25th, 50th, and 75th percentile of total energy intake from left to right and have been adjusted for age, sex, household income, BMI, and physical activity.

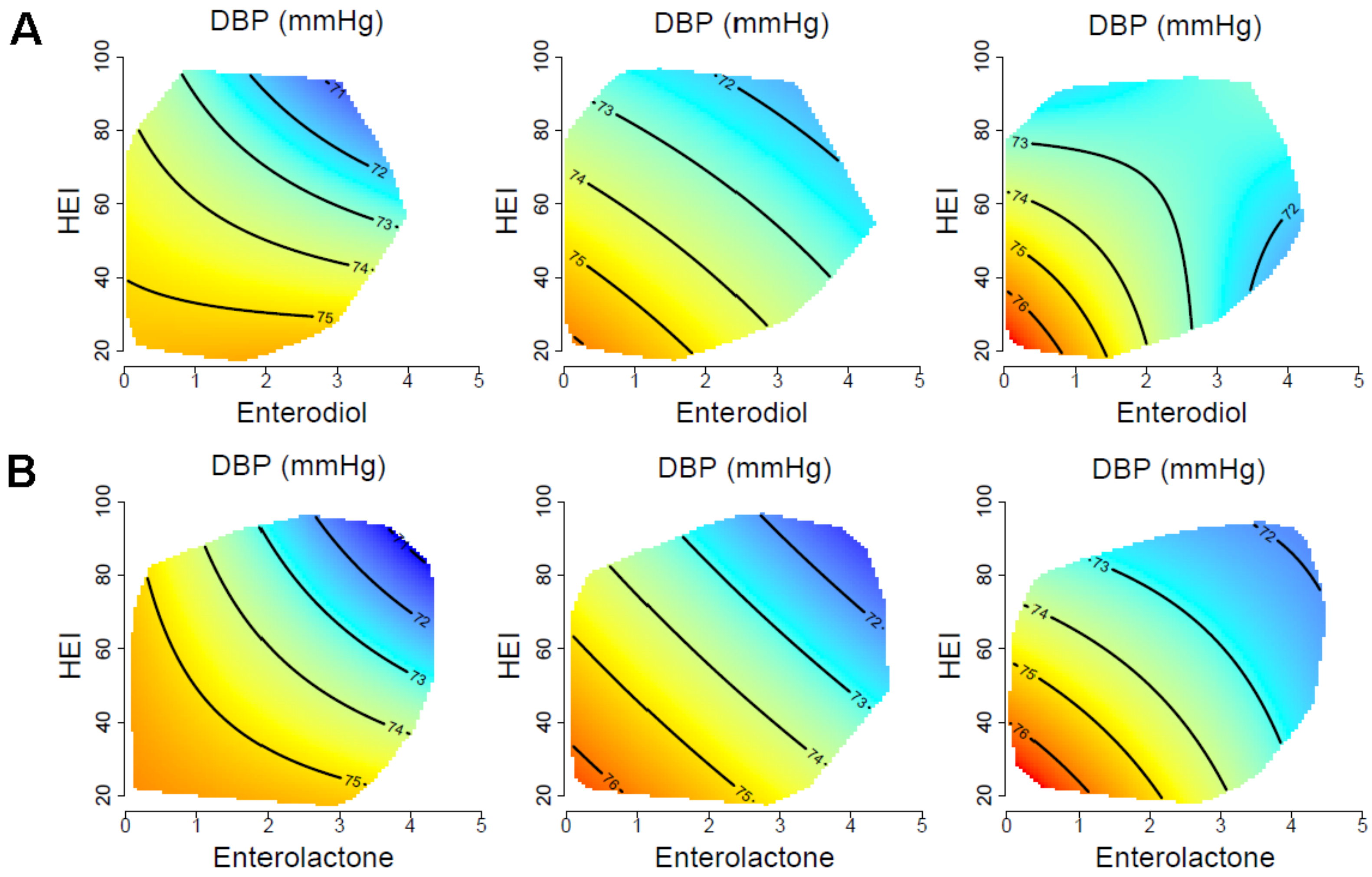

Figure S14. Associations of Diastolic Blood Pressure with HEI, Enterolignans, and Energy Intake. Response surfaces show the associations of diastolic blood pressure with HEI, enterodiol (A), enterolactone (B), and total energy intake. Enterolactone and enterodiol are presented as µmol/L (log-transformed). The outcome of each response surface is shown at the top of the plot with warmer colors denoting higher values and cooler colors denoting lower values. Response surfaces are predicted at the 25th, 50th, and 75th percentile of total energy intake from left to right and have been adjusted for age, sex, household income, BMI, and physical activity.

Table S1. Model Coefficients for Varying Levels of Interaction for Cardiometabolic Health, Diet Quality, and Microbial Lignan Metabolites[1]

		Model[1]			*Model[2]*			*Model[3]*		
**Outcome**	**Metabolite**	***DE***	***AIC***	***P***	***DE***	***AIC***	***P***	***DE***	***AIC***	***P***
Triglycerides	Enterodiol	16.1%	25,575.4	0.03	15.8%	25,590.4	0.02	16.7%	25,536.6	0.001
	Enterolactone	16.7%	25,546.4	<0.001	16.7%	25,546.4	<0.001	17.4%	25,501.2	<0.001
Total Cholesterol	Enterodiol	13.7%	40,317.1	0.60	13.6%	40,317.3	0.48	13.8%	40,321.9	0.48
	Enterolactone	13.8%	40,313.6	0.15	13.7%	40,315.5	0.14	13.8%	40,320.4	0.37
LDL Cholesterol	Enterodiol	12.7%	21,063.5	0.32	12.6%	21,065.2	0.52	12.9%	21,070.0	0.009
	Enterolactone	12.7%	21,064.2	0.44	12.6%	21,065.5	0.76	12.8%	21,072.5	0.02
HDL Cholesterol	Enterodiol	24.8%	35,529.4	0.002	24.6%	35,527.9	<0.001	24.8%	35,531.5	<0.001
	Enterolactone	24.6%	35,533.0	0.004	24.5%	35,534.6	0.001	24.7%	35,537.3	<0.001
Glucose	Enterodiol	15.6%	19,110.0	0.08	15.6%	19,110.0	0.07	15.9%	19,111.0	0.12
	Enterolactone	15.4%	19,118.7	0.70	15.4%	19,118.7	0.61	15.7%	19,122.3	0.36
Insulin	Enterodiol	34.7%	14,062.4	0.31	34.7%	14,062.4	0.31	34.9%	14,061.6	0.03
	Enterolactone	34.8%	14,062.5	0.13	34.8%	14,062.8	0.11	35.1%	14061.4	0.007
OGTT	Enterodiol	20.8%	10,281.1	0.02	20.8%	10,281.1	0.02	21.6%	10,277.0	0.02
	Enterolactone	20.5%	10,287.1	0.09	20.5%	10,287.1	0.09	21.1%	10,289.2	0.07
HbA1c	Enterodiol	15.0%	4,713.3	0.002	15.0%	4,713.3	0.002	15.0%	4,723.4	0.21
	Enterolactone	14.6%	4,726.9	0.19	14.6%	4,726.9	0.19	14.7%	4,738.1	0.90
Body Fat (%)	Enterodiol	44.2%	7,930.1	0.70	44.2%	7,930.1	0.70	44.5%	7,935.6	0.02
	Enterolactone	44.4%	7,926.5	0.04	44.4%	7,926.5	0.04	44.6%	7,933.8	0.01
Systolic Blood Pressure	Enterodiol	31.3%	36,771.9	<0.001	31.6%	36,774.2	0.11	31.4%	36,778.3	<0.001
	Enterolactone	31.7%	36,750.9	<0.001	32.0%	36,753.6	<0.001	31.8%	36,754.7	<0.001
Diastolic Blood Pressure	Enterodiol	12.8%	34,669.2	0.02	12.8%	34,669.2	0.02	12.8%	34,675.9	0.007
	Enterolactone	12.8%	34,675.1	0.01	12.8%	34,667.5	0.004	12.8%	34,675.1	0.003

[1]P-value reflects the level of significance for enterolignans across increasingly complex models for the outcome variable (triglycerides, total cholesterol, low-density lipoprotein [LDL] cholesterol; high-density lipoprotein [HDL] cholesterol; systolic blood pressure; diastolic blood pressure; body fat percentage; glucose; insulin; oral glucose tolerance test [OGTT]; hemoglobin A1c [HbA1c]). Models with the most favorable Akaike information criterion (AIC) value are shaded gold. Multiple models were highlighted in rows where there was no material difference in AIC (>2 points). The percentage of deviance explained (DE) is for the entire model.

Model[1] – Individual additive model: s(enterolignan) + s(diet quality) + s(kcal)

Model[2] – Two-way interactive model: s(enterolignan, diet quality) + s(kcal)

Model[3] – Three-way interactive model: s(enterolignan, diet quality, kcal)

Table S2. Model Coefficients for Waist Circumference Sensitivity Analysis[1]

		Model Coefficients		
**Outcome**	**Metabolite**	***DE***	***AIC***	***P***
Triglycerides	Enterodiol	18.8%	25,066.48	0.006
	Enterolactone	19.7%	25,019.54	<0.001
Total Cholesterol	Enterodiol	14.4%	39,369.87	0.27
	Enterolactone	14.3%	39,369.75	0.25
LDL Cholesterol	Enterodiol	13.7%	20,655.57	0.02
	Enterolactone	13.6%	20,659.74	0.07
HDL Cholesterol	Enterodiol	26.7%	34,617.59	<0.001
	Enterolactone	26.6%	34,622.71	<0.001
Glucose	Enterodiol	17.3%	18,706.93	0.24
	Enterolactone	17.2%	18,714.57	0.44
Insulin	Enterodiol	36.5%	13,634.98	0.04
	Enterolactone	36.6%	13,632.29	0.009
OGTT	Enterodiol	17.3%	18,706.93	0.24
	Enterolactone	22.1%	10,184.26	0.12
HbA1c	Enterodiol	15.2%	4,616.49	0.32
	Enterolactone	14.9%	4,628.93	0.93
Body Fat (%)	Enterodiol	44.7%	7,938.05	0.007
	Enterolactone	44.6%	7,940.32	0.02
Systolic Blood Pressure	Enterodiol	31.2%	36,108.52	<0.001
	Enterolactone	31.4%	36,091.9	<0.001
Diastolic Blood Pressure	Enterodiol	13.7%	34,048.16	0.01
	Enterolactone	13.7%	34,051.03	0.03

[1]P-value reflects the level of significance for microbial lignan metabolites, HEI score, and total energy intake as a three-dimensional smooth term for the outcome variable (triglycerides, total cholesterol, low-density lipoprotein [LDL] cholesterol; high-density lipoprotein [HDL] cholesterol; systolic blood pressure; diastolic blood pressure; body fat percentage; glucose; insulin; oral glucose tolerance test [OGTT]; hemoglobin A1c [HbA1c]). This model represents the fully adjusted model with waist circumference adjustment instead of BMI. Akaike information criterion scores (AIC) are shown as an indicator of model fitness where lower values have a better overall fit. A difference in AIC of >2 was considered evidence of a difference in model fit. Deviance explained (DE) is shown for the entire specified model. Body fat percentage was not adjusted for waist circumference.

Table S3. Male Stratified Model Coefficients for Cardiometabolic Health, Diet Quality, and Microbial Lignan Metabolites[1]

		Model[1]			*Model[2]*			*Model[3]*		
**Outcome**	**Metabolite**	***DE***	***AIC***	***P***	***DE***	***AIC***	***P***	***DE***	***AIC***	***P***
Triglycerides	Enterodiol	9.4%	13,343.6	<0.001	11.8%	13,313.9	<0.001	18.3%	13,244.1	<0.001
	Enterolactone	9.4%	13,359.3	<0.001	11.9%	13,326.0	<0.001	18.0%	13,258.0	0.001
Total Cholesterol	Enterodiol	10.1%	20,363.2	0.48	10.1%	20,366.5	0.45	13.0%	20,313.0	0.75
	Enterolactone	10.1%	20,362.7	0.42	10.2%	20,366.0	0.40	13.0%	20,312.2	0.66
LDL Cholesterol	Enterodiol	9.1%	10,568.1	0.09	9.1%	10,571.8	0.13	12.7%	10,542.1	0.10
	Enterolactone	8.9%	10,569.9	0.15	9.0%	10,573.4	0.20	12.6%	10,542.8	0.13
HDL Cholesterol	Enterodiol	3.4%	17,522.5	<0.001	5.1%	17,486.7	<0.001	17.4%	17,189.9	<0.001
	Enterolactone	3.1%	17,529.0	<0.001	4.8%	17,492.2	<0.001	17.1%	17,198.5	0.002
Glucose	Enterodiol	11.0%	9,634.2	0.01	12.2%	9,620.4	0.01	15.1%	9,593.7	0.02
	Enterolactone	10.2%	9,648.8	0.08	11.5%	9,634.1	0.07	14.4%	9,608.1	0.15
Insulin	Enterodiol	2.2%	7,691.7	0.22	3.0%	7,690.0	0.24	37.1%	7,132.8	0.39
	Enterolactone	3.4%	7,676.8	0.008	4.1%	7,677.7	0.01	37.2%	7,133.4	0.26
OGTT	Enterodiol	15.2%	5,276.7	0.12	16.2%	5,272.1	0.13	20.0%	5,259.8	0.12
	Enterolactone	14.7%	5,285.5	0.19	15.6%	5,282.0	0.23	19.3%	5,270.8	0.26
HbA1c	Enterodiol	8.7%	2,354.2	0.002	9.6%	2,343.8	0.004	12.3%	2,311.0	0.007
	Enterolactone	8.0%	2,368.1	0.03	8.9%	2,358.01	0.06	11.4%	2,328.0	0.15
Body Fat (%)	Enterodiol	6.0%	3,898.2	0.17	6.2%	3,900.47	0.12	19.7%	3,820.2	0.54
	Enterolactone	6.0%	3,897.7	0.14	6.4%	3,899.6	0.09	20.0%	3,818.7	0.32
Systolic Blood Pressure	Enterodiol	18.6%	18,266.9	0.007	19.0%	18,261.0	0.01	21.2%	18,215.3	0.04
	Enterolactone	19.1%	18,254.5	<0.001	19.4%	18,249.4	<0.001	21.5%	18,207.2	0.003
Diastolic Blood Pressure	Enterodiol	10.9%	17,458.9	0.07	11.4%	17,451.1	0.07	12.9%	17,424.0	0.12
	Enterolactone	11.1%	17,455.5	0.02	11.5%	17,449.2	0.03	12.9%	17,423.9	0.09

[1]P-value reflects the level of significance for microbial lignan metabolites, HEI score, and total energy intake as a three-dimensional smooth term for the outcome variable (triglycerides, total cholesterol, low-density lipoprotein [LDL] cholesterol; high-density lipoprotein [HDL] cholesterol; systolic blood pressure; diastolic blood pressure; body fat percentage; glucose; insulin; oral glucose tolerance test [OGTT]; hemoglobin A1c [HbA1c]). Akaike information criterion (AIC) and percentage of deviance explained (DE) are shown for the entire model. Body fat percentage was not adjusted for BMI in Model[3].

Model[1]: Adjusted for Age, Household Income

Model[2]: Adjustments as per model 1 + Race/Ethnicity + Education Level

Model[3]: Adjustments as per model 2 + Smoking + Alcohol + Physical Activity + BMI

Table S4. Female Stratified Model Coefficients for Cardiometabolic Health, Diet Quality, and Microbial Lignan Metabolites[1]

		Model[1]			*Model[2]*			*Model[3]*		
**Outcome**	**Metabolite**	***DE***	***AIC***	***P***	***DE***	***AIC***	***P***	***DE***	***AIC***	***P***
Triglycerides	Enterodiol	7.0%	12,266.2	0.41	9.6%	12,252.7	0.43	15.6%	12,185.1	0.76
	Enterolactone	8.4%	12,237.0	0.007	11.5%	12,213.2	0.001	17.2%	12,145.9	0.008
Total Cholesterol	Enterodiol	17.5%	19,963.4	0.50	17.5%	19,966.3	0.50	18.9%	19,946.8	0.52
	Enterolactone	17.6%	19,961.4	0.36	17.6%	19,963.8	0.32	19.0%	19,943.6	0.30
LDL Cholesterol	Enterodiol	12.2%	10,549.2	0.25	12.5%	10,550.0	0.18	15.0%	10,533.1	0.38
	Enterolactone	17.6%	19,961.9	0.36	12.5%	10,550.0	0.18	15.1%	10,532.9	0.37
HDL Cholesterol	Enterodiol	7.9%	18,592.4	<0.001	10.0%	18,540.8	<0.001	20.8%	18,269.0	<0.001
	Enterolactone	8.3%	18,583.5	<0.001	10.5%	18,530.1	<0.001	20.9%	18,264.5	<0.001
Glucose	Enterodiol	14.4%	9,539.1	0.06	16.0%	9,512.7	0.12	19.8%	9,471.2	0.19
	Enterolactone	14.1%	9,544.3	0.14	15.7%	9,516.2	0.22	19.7%	9,471.5	0.25
Insulin	Enterodiol	6.6%	7,319.0	<0.001	7.7%	7,315.1	<0.001	38.9%	6,815.2	<0.001
	Enterolactone	12.2%	10,549.2	0.25	8.6%	7,299.5	<0.001	39.2%	6,807.9	<0.001
OGTT	Enterodiol	19.7%	5,056.1	0.20	20.9%	5,052.2	0.19	28.6%	5,007.2	0.16
	Enterolactone	20.0%	5,053.8	0.12	21.2%	5,050.0	0.13	28.6%	5,007.7	0.18
HbA1c	Enterodiol	14.7%	2,490.1	0.88	15.6%	2,476.8	0.88	22.1%	2,370.3	0.95
	Enterolactone	14.9%	2,487.1	0.66	15.8%	24,73.94	0.67	22.3%	2,365.2	0.66
Body Fat (%)	Enterodiol	5.8%	3,861.9	0.24	5.8%	3,865.7	0.26	39.5%	3,633.4	0.42
	Enterolactone	6.3%	3,858.8	0.09	6.3%	3,862.7	0.09	39.8%	3,630.26	0.17
Systolic Blood Pressure	Enterodiol	38.3%	18,498.6	0.002	38.4%	18,500.1	0.005	40.6%	18,421.7	0.04
	Enterolactone	38.6%	18,486.8	<0.001	38.7%	18,488.7	<0.001	40.8%	18,423.2	0.10
Diastolic Blood Pressure	Enterodiol	10.5%	17,267.6	0.009	11.0%	17,258.7	0.006	12.0%	17,239.7	0.03
	Enterolactone	20.0%	5,053.8	0.12	10.9%	17,260.8	0.01	11.9%	17,241.6	0.04

[1]P-value reflects the level of significance for microbial lignan metabolites, HEI score, and total energy intake as a three-dimensional smooth term for the outcome variable (triglycerides, total cholesterol, low-density lipoprotein [LDL] cholesterol; high-density lipoprotein [HDL] cholesterol; systolic blood pressure; diastolic blood pressure; body fat percentage; glucose; insulin; oral glucose tolerance test [OGTT]; hemoglobin A1c [HbA1c]). Akaike information criterion (AIC) and percentage of deviance explained (DE) are shown for the entire model. Body fat percentage was not adjusted for BMI in Model[3].

Model[1]: Adjusted for Age, Household Income

Model[2]: Adjustments as per model 1 + Race/Ethnicity + Education Level

Model[3]: Adjustments as per model 2 + Smoking + Alcohol + Physical Activity + BMI

Table S5. Model Coefficients for Cardiometabolic Health, Diet Quality, and Microbial Lignan Metabolites with Interaction by Sex[1]

		Male			*Female*		
**Outcome**	**Metabolite**	***DE***	***AIC***	***P***	***DE***	***AIC***	***P***
Triglycerides	Enterodiol	17.8%	25,499.24	<0.001	17.8%	25,499.24	0.93
	Enterolactone	18.3%	25,479.12	<0.001	18.3%	25,479.12	0.18
Total Cholesterol	Enterodiol	14.2%	40,328.32	0.21	14.2%	40,328.32	0.29
	Enterolactone	14.0%	40,328.41	0.26	14.0%	40,328.41	0.50
LDL Cholesterol	Enterodiol	13.1%	21,082.49	0.02	13.1%	21,082.49	0.49
	Enterolactone	13.0%	21,084.39	0.04	13.0%	21,084.39	0.56
HDL Cholesterol	Enterodiol	25.0%	35,537.93	<0.001	25.0%	35,537.93	<0.001
	Enterolactone	25.0%	35,544.17	0.02	25.0%	35,544.17	<0.001
Glucose	Enterodiol	17.0%	19,092.84	0.03	17.0%	19,092.84	0.12
	Enterolactone	16.6%	19,109.14	0.16	16.6%	19,109.14	0.19
Insulin	Enterodiol	35.3%	14,051.89	0.31	35.3%	14,051.89	0.02
	Enterolactone	35.6%	14,040.41	0.11	35.6%	14,040.41	0.005
OGTT	Enterodiol	22.5%	10,279.43	0.02	22.5%	10,279.43	0.37
	Enterolactone	22.0%	10,294.15	0.08	22.0%	10,294.15	0.41
HbA1c	Enterodiol	15.7%	4,719.16	0.006	15.7%	4,719.16	0.89
	Enterolactone	15.4%	4,730.82	0.10	15.4%	4,730.82	0.66
Body Fat (%)	Enterodiol	44.7%	7,946.66	0.04	44.7%	7,946.66	0.57
	Enterolactone	44.9%	7,942.89	0.02	44.9%	7,942.89	0.33
Systolic Blood Pressure	Enterodiol	31.5%	36,790.82	0.004	31.5%	36,790.82	0.08
	Enterolactone	32.1%	36,765.71	<0.001	32.1%	36,765.71	0.03
Diastolic Blood Pressure	Enterodiol	13.0%	34,682.57	0.08	13.0%	34,682.57	0.02
	Enterolactone	13.2%	34,680.05	0.02	13.2%	34,680.05	0.06

[1]Model outputs for associations of macronutrient intake with cardiometabolic health markers, microbial lignan metabolites, and diet quality with an interaction term for sex in the fully adjusted model. Akaike information criterion scores (AIC) are shown as an indicator of model fitness where lower values have a better overall fit. A difference in AIC of >2 was considered evidence of a difference in model fit. Highlighted rows denote a significant improvement in AIC compared to the model with no sex interaction term. The percentage of deviance explained (DE) is for the entire model. Low-density lipoprotein (LDL) cholesterol; high-density lipoprotein (HDL) cholesterol; oral glucose tolerance test (OGTT); hemoglobin A1c (HbA1c).
